# Tradesmen's Books

**Published:** 1908-08-15

**Authors:** 


### Nursing Administration.

### TRADESMEN'S BOOKS.

The slipshod ways of the ordinary tradesman or contractor are known only too well to housekeepers. Half the difficulty in making out the ordinary week's expenditure on food starts from slovenly bookkeeping outside. Going through the books is a process every mistress of a house has reason to dread, and in an institution this business is more than an irksome task —it is a heavy responsibility. The smallest deviation from accuracy is a matter of importance, and the effort to bring order into a muddled array of facts and figures consumes many weary hours. As usually presented, the invoices and books of tradesmen are an interesting example of "how not to do it." Legibility being the first desideratum, these sheets are commonly presented in grotesque and highly abbreviated scrawls. As, for instance, the butcher, instead of writing plain English, claims in hideous hieroglyphics to have supplied r.st. and kdny., trgt. lb., or slvsde. Again, the price charged is a detail of some importance, and the housekeeper, even where this has been settled by contract, will like to be sure she is being served at the correct figure. But the only clue to the price is the total cost of the joint, and if it is desired to know how much the butcher has charged per pound, it is necessary to reckon it out by some such process as dividing 5s. 11½d. by 5 lbs. 15 oz. All this is thoroughly unsatisfactory. The large institutions make their own stipulations as to the way in which accounts and invoices should be presented. And it is in the power of even the smallest establishment to have their accounts simplified by arranging for a uniform system of tradesmen's books, and thus to bring order into this neglected branch of household economy. From the housekeeper's point of view the important thing to have clearly set forth in the book is the amount supplied every day, whether the commodity be meat, bread, milk, vegetables, or any other perishable article not kept in stock. We append a specimen leaf from the book of a well-known dairy company, which is admirably calculated to facilitate good book-

Week ending.....................................190.........

| | Sun. | Mon. | Tue. | Wed. | Thu. | Fri. | Sat. | Total | At | £ s. d. |
|---|---|---|---|---|---|---|---|---|---|---|
| Milk ... ... | | | | | | | | | | |
| Cream... ... | | | | | | | | | | |
| Butter, fresh... | | | | | | | | | | |
| " salt ... | | | | | | | | | | |
| Eggs, new laid | | | | | | | | | | |
| " cooking | | | | | | | | | | |
| Week's total .. ... | | | | | | | | | | |
| Brought forward ... | | | | | | | | | | |
| Total ... ... | | | | | | | | | | |

keeping in any establishment. It will be seen that the amount daily supplied is specified, but the price remaining constant, each day's cost is immaterial, and the cost for the week's supply is reckoned at the end. The housekeeper at a glance is able to tell the smallest variation in the supply, and to check instantly the figures from her own similar book of quantities received. Whether payments are made weekly, monthly, or quarterly, she has a clear picture of the week's consumption and its cost, and were this system carried right through all the other books, she would hardly need any other guide than her weekly bills to make out the average cost of each person's board in the establishment. True, the list of articles supplied would be a longer one in the case of the butcher and greengrocer, but the ordinary articles of consumption vary little in institutions, and space can be left for writing in such things as vary with the season. The cost of preparing books on this system is trifling, and would be readily borne by the supplier, if the desired system were explained.

In dealing with the grocer or stores for articles kept in stock a slight variation is necessary. No one can calculate beforehand how much semolina or rice, or sugar will be needed in a week. These things are commonly ordered in bulk, once a month or once a quarter, and are given out from the storeroom. For the purpose of the weekly calculation, the storeroom must reckon as a shop, and the housekeeper, in measuring and weighing out the stores every day, must record each transaction in a book ruled to match the above specimen. Against each article each day she enters the quantity issued, and only at the end of the week does she calculate from her book of prices the cost for the week. Thus the grocer's book in this form is available to make up the total of the week's expenditure. In order to separate the cost of the patients' food from that of the staff, it is convenient in giving out stores to enter articles intended solely for the patients in a separate book. The diet sheets show precisely the weight of bread, meat, fish, etc., consumed by the patients; these amounts are deducted, and thus, with the smallest expenditure of time and labour, the cost of the staff can be disentangled and set forth week by week. We commend this system to matrons of small establishments, where facilities are not at hand for keeping the cost of the patients entirely distinct from that of the staff and household, and where it is desired to work out the weekly cost of each member of the household in an expeditious manner. With her pile of orderly tradesmen's books before her, the matron has more than half the labour of computation already accomplished. She has merely to obtain the weekly average of patients, and deduct in accordance with the diet table the amounts assigned to them. Then, adding the total of all the books, and including her own stores, she divides by the weekly average of persons boarding in the house, and enters the figures in a book kept for the inspection of the house committee. Those housekeepers who have practised keeping their eye on the variations of weekly expenditure per head have found it an immense source of strength in practising economy, and in giving a firm grip over the commissariat. Not the least of its advantages is the power it gives of experimenting in various directions to lessen the deadly monotony of institution housekeeping.

